# Cognitive Strategies and Textual Genres in the Teaching and Evaluation of Advanced Reading Comprehension (ARC)

**DOI:** 10.3389/fpsyg.2021.723281

**Published:** 2021-10-12

**Authors:** Jesús-Nicasio García-Sánchez, Judit García-Martín

**Affiliations:** ^1^Department of Psychology, Sociology and Philosophy, Universidad de León, León, Spain; ^2^Department of Humanities, Universidad De La Costa, Barranquilla, Colombia; ^3^Department of Developmental and Educational Psychology, Universidad de Salamanca, Salamanca, Spain

**Keywords:** advanced reading comprehension, instruction strategies, assessment systems, generic competences across curriculum, higher education

## Abstract

In the last decade, published data on the performance of Colombian students have concerned educators and researchers, making critical reading one of the priorities of Colombian education. That is why this article presents the results of a study carried out in a Latin American university in which the perceptions of students and professors are analyzed regarding the strategies and textual genres used to work and cross-evaluate the advanced reading comprehension (ARC). This study is materialized in the application of an ad *hoc* online questionnaire, in its two versions (students and teachers), designed through Survey Monkey. For this, it has the participation of 182 teachers and 2,775 students. There are several trends in the use of specific textual strategies and typologies to work and evaluate ARC, by both, depending on the department of assignment. The evidence found is provided and evaluated considering the implications for cross-curricular instruction and assessment in higher education in Latin America, including study limitations and prospects for overcoming them.

## Introduction

Government agencies, in order to comply with the standards of the Ministry of Education, publish, every 3 years, the results obtained in the international PISA tests, regarding the performance of students in different areas of knowledge, among which are, reading (OCDE, 2016). In this sense, in the last decade, published data on the performance of Colombian students have concerned educators and researchers (Díaz et al., 2015; García J. N. et al., 2019; García J. R. et al., 2019; Inciarte et al., 2019; Marín et al., 2019; Rueda et al., 2019). That is why critical reading or advanced reading comprehension has become one of the priorities of Colombian education (Cassany, 2003; Cubides et al., 2017).

In this sense, in 2003, Cassany affirms that critical reading or advanced reading comprehension (hereinafter, ARC) is an explicit approximation of a demanding and complex reading typology that apart from the essential requirements of literal, inferential and intentional of the text, requires higher demands, as well as the interest expressed by the reader's understanding. All this implies implicitly the recovery of the connotations of the words used, the identification of the author's point of view, with special attention to the use of sarcasm and irony…, the distinction between the voices used and of course the identification of the textual genre employed in speech. In line with the above, in the Colombian higher education space, the mandatory national test, *Saber Pro*, is the most important source of quality to examine five modules: (i) critical reading, (ii) quantitative reasoning, (iii) citizen competence, (iv) written communication and (v) English (García J. N. et al., 2019; García J. R. et al., 2019; Cabeza et al., 2020; Calderón et al., 2021). According to Calderón et al. (2021), the module related to critical reading has a total of 35 questions that evaluate three types of comprehension: (i) literal and explicit, in terms of the meaning of words and expressions used. and that represents 25% of the test, (ii) the global one that involves 40% of the questions and (iii) the criticism that covers 35% of the questions posed and that is based on the recognition of the strategies used, the identification of the different assumptions, the validity of the arguments….

The role that advanced reading comprehension plays in the rest of the generic and specific competences is essential. For example, the quality of the written composition (written communicative competence) are designed from the planning processes that involve the generation of information and ideas, or the selection of ideas and review of the written message, so that reading comprehension becomes essential. (García and García-Martín, 2021a,b; Graham, 2021; López et al., 2021; Robledo-Ramón and García-Gutiérrez, 2021). The same happens with citizen competence, where critical thinking represents 70% of the assessment of competence, which implies the resolution of citizen problems, which require a high dose of advanced reading comprehension, as in the case of the development of lateral reading strategies and contrast of facts in the face of hoaxes and fakes (Brodsky et al., 2021; Cabrera et al., 2021). It is evident that advanced reading comprehension plays a role in generic quantitative reasoning competence. Solving computational problems, which involves quantification, owes much to the mastery of advanced reading comprehension. Evidence from the analysis of the semester tests of generic competences in the reference institution, as well as the analysis of more than a decade of the big data available in the ICFES of the MEN of Colombia, indicate that reading comprehension is essential for the quantitative competence (and general intelligence), and others such as English. It is evident that he also plays it for the different specific competences, typical of each profession and program or career (García and Jiménez, 2017; García-Martín and García-Sánchez, 2020). For example, the national Saber Pro test involves three types of components for the assessment of quantitative reasoning competence: interpretation and representation; formulation and execution; and argumentation. In all three components, advanced reading comprehension is essential; in addition to asking contextual or situational questions, both family and personal, work or occupational; social or community; and in that of scientific dissemination (Calderón et al., 2021). A competency, also transversal and generic, that is gaining more importance every day, is digital, for which high doses of advanced reading comprehension are also required and which plays a key role in enhancing the rest of the generic competences and specific, in addition to any higher learning (García-Martín and García-Martín, 2021).

### Cognitive Strategies and Textual Genres for the Teaching and Assessment of ARC

Higher Education Institutions in Colombia focus on examining the quality of the processes used (Orozco, 2010; Arias et al., 2018). In this sense, Colombian universities, as the focus of this study, are presented as higher education institutions that promote cognitive strategies and textual genres that enhance the transversal development-through the curriculum-of critical reading or advanced reading comprehension of the students (Samper and Ospino, 2018), making them competent to respond to the needs and demands of today's multicultural, globalized, and dynamic society (Barnet, 2001; Castellar et al., 2021).

On the one hand, the review of previous studies and antecedents allowed to identify the different components, processes, strategies, genres, and instructional means involved in the evaluation and teaching of advanced reading comprehension. From the perspective of cognitive psychology, for example, the psychology of reading, it seems reasonable to consider processes, techniques and strategies related to the activation of previous knowledge, the relationship of meaning between elements, the ability to draw inferences, generation of mental models that allow the representation of the meaning of the text, the ability to apply meanings to other areas of knowledge and life; as well as the structure reflected through the most common textual genres in the university environment that are part of the textual macrostructure (argumentative, comparison and contrast, cause and effect, problem solution); and finally, the means deployed to access textual information, be it traditional or virtual.

From the model that is deployed in the *Saber Pro* tests (Calderón et al., 2021); and on the other, from the broad tradition of cognitive psychology, instructional psychology, and scientific psychology in general, many instruments, empirical investigations, contrasted evidence in instructional or intervention studies were analyzed, it was possible to construct the instruments used in this study research (García-Martín and García-Sánchez, 2020). The interest on the subject is reflected in the systematic reviews that analyze reading and writing through content (literacy across content) in which it seems necessary to answer questions about the moment, the procedures, the actions, in relation to the introduction from the beginning. assessment and teaching of advanced reading comprehension in the different disciplines and subjects of all university degrees. But, in addition, these reviews show the scarcity of studies that provide empirical validation. The example from the Scott and Washburn (2018) is paradigmatic. After analyzing 50 years of applications of literacy across content in the training of teachers in the USA, after contrasting its methodological quality, only twenty-nine studies can be included, and of these, only four allowed a quantitative comparison and therefore generalizable with other studies. The foci of what they perceive to be happening, of resistance to changes, and of the experience deployed in the training of new teachers, seem to be the key variables in this story. Along the same lines is the systematic review by Miller et al. (2018). This situation in Europe is not more encouraging, as the review by Uttl et al. (2017) refers to, making a meta-analysis of the previous meta-analyzes on the evaluation of teachers by the perceptions of their students about their satisfaction with the evaluation and teaching, a common strategy in the different universities of the world, is disappointing, since, ultimately, the evidence provided by the empirical studies analyzed depends on the size of the samples, since with large samples no effects are found (that is why there are no only published studies), and yes with small samples (published studies). It is necessary to recognize the need to implement different strategies to verify the role of evaluation systems and instructional techniques, strategies, procedures, and processes in the deployment of generic competences in a transversal way in all subjects of the degree. The perception of the level of satisfaction with the evaluation (Reyes et al., 2020), or of the teaching methods used (Sánchez et al., 2019; Jiménez et al., 2020), or the types of actions deployed with advanced reading comprehension (Valero et al., 2015) or even the type of strategies such as the narratives deployed (Del Moral-Pérez et al., 2016) seem to mark relevant focuses and variables that must be analyzed, In the cultural field Asian, reflects the same trend. For example, the systematic review by Li et al. (2018) finds fifty-nine studies in Mainland China that meet the inclusion and exclusion criteria published during the previous 20 years, focused on constructs and focuses related to self-regulation of learning, finding as key variables the beliefs of capacity with learning, instructional strategies and the capacity for self-awareness and self-evaluation of instructional processes as a guarantee of self-regulatory capacity for productive and effective learning. Of these fifty-nine studies, only four provide data that allow comparative calculations, with two or more groups: the rest being only pre-post comparisons. The need to identify the variables responsible for the improvement of generic competences seems evident. And this is what contributes, also the impressive review of Peng et al. (2019) in which they find about fifty thousand studies, of which six hundred fifty meet the inclusion and exclusion criteria that they analyze with the meta-analysis methodology, referred to the role of fluid and crystallized intelligence in math instruction and reading comprehension. The above limitations are confirmed, in addition to the need to study evaluation and instruction variables, psychoeducational and instructional variables, such as those analyzed in this study.

### Research Context and Theoretical Axes

It is a general project of analysis and promotion of the mainstreaming of generic competences or through subjects (García J. N. et al., 2019; García J. R. et al., 2019). The latest development has led to the creation of a skills observatory, initially focusing on generic skills, but with a view to including specific skills as well (García-Martín and García-Sánchez, 2020). On the one hand, this underlies, supports and covers the national final career tests of Saber Pro (Calderón et al., 2021), and on the other, the internal semester tests that are similar to the previous ones, but that affect the diagnosis from the first semester of each career, the state of the domain of generic and specific competences of all students, in order to strengthen and enhance them through actions designed in the evaluation and teaching of each subject (Sánchez et al., 2019). Within this framework, studies have been implemented on the teaching and evaluation of generic competences of advanced reading comprehension, written communication and, last year, on civic competence (with very large components of advanced reading comprehension, in addition to critical thinking), along with other psychoeducational variables, such as self-efficacy, emotional intelligence and social performance, coping strategies in the face of problems, attitudes and anxiety in the face of these competences, among others, including mastery of the digital competence (García-Martín and García-Sánchez, Submitted). From the institutional perspective and high quality accreditation, the creation of the competences observatory is an unbeatable research context, in addition to serving as a catalyst and catalyst for the design of research, initiatives, innovations, promoting the quality of teaching, through the mainstreaming of competences in all subjects of all careers (CUC, 2018; García and García-Martín, 2021a,b).

The study's frame of reference focuses on five clearly interwoven and integrated axes (Marín et al., 2019). First, competency-focused teaching and the EHEA (European Higher Education Area), for the specific case of Colombia, the OCDE report is essential for understand it (OCDE, 2016). Second, active university methodologies toward a ubiquitous web 4.0. The latest advances regarding the introduction of active methodologies and innovations in university teaching are key, including advances and studies with digital competence, and the so-called web 4.0 (García-Martín and García-Sánchez, Submitted). In addition, a psychological and curricular instructional perspective. The instructional advances and curricular innovations are undeniable, both coming from the psychological, educational and contextual disciplines, which provide a first-line frame of reference for promoting the learning of competencies in the different subjects, including the psychoeducational variables, which are key in this process. (García-Martín and García-Sánchez, 2020; García-Martín and García-Martín, 2021). Fourth, the latest developments in instrument validation technology. The validation of instruments is key in any process of a didactic nature or of investigation. In the project of Evaluation and Methodologies and Incidents in Competences Generic (EMICOG) aimed at studying teaching methodologies and forms of evaluation cross-curricular subjects, from its rigorous and scientific nature, it is necessary to design, validation and application of instruments that in a standardized way allow the collection appropriate information (Chen and Lin, 2018; Cuesta et al., 2018; Habók and Magyar, 2018; Martínez-Ferrer et al., 2018; Puente-Martínez et al., 2018; Romera et al., 2018; Sinval et al., 2018; Zeng et al., 2019). And finally, the perspective of empirically-based interventions and quality indicators of innovations. The practices based on scientific evidence or empirically validated, have been the basis for selecting innovations that work, that are successful, and that should be promoted and promoted. This is the case of the best practices of the USOE (2021), or of the guidelines, protocols, validated programs promoted by the CEEDAR (2021).

### Research Question

In line with the above, the *research question* of this study is: what does the analyze of techniques, strategies, cognitive processes contribute; as well as the textual genres used in teaching and in the cross-sectional evaluation of critical reading or advanced reading comprehension, in a Latin American university, from a double perspective: that of the teaching staff and that of the students? This raises various hypotheses, on the one hand, it is expected that teachers show a greater knowledge of cognitive strategies and textual genres to teach and evaluate ARC than students, it is expected that there are differential patterns in the use of cognitive strategies and textual genres to teach and evaluate the ARC by teachers and students, according to the department to which they are attached.

## Method

### Participants

The sample of this study is made up of 203 teachers (125 men and 78 women) aged between 24 and 73 years with a mean age of 41 years and 2,775 students (1,336 men and 1,439 women) with a mean age of 22 years. The collaboration of all the students and all the professors of the institution was requested. For professors 525 permanent (40% international) and 204 non-permanent. 280 agreed to participate, although 203 answered all the questions. For the students, 3,645 agreed to collaborate, although with the complete answers 2,775, which represents ~30% of the total of students in all grades and semesters. With a simple random sampling, confidence level of 99% and margin of error of 1% (CUC, 2018; García-Martín and García-Sánchez, 2020; García and García-Martín, 2021a,b). This guarantees great diversity and representativeness to be considered a high-quality type institution in Latin America. All of them assigned to eight departments (Computer Science, Economics, Exact, Environmental, Law, Energy, Industrial and Humanities) of the focal university of study, which decide to participate in an informed and voluntary way, through the completion of the ad *hoc* online questionnaire, EMICOG. The departments included in the sampling and analysis are the eight academic and specific, since the University Extension is transversal and for the purposes of teacher training, as well as for the promotion of the mainstreaming of competences, among other functions. The data of all the participants are checked and verified (see Table 1), being representative of a high quality accredited Latin American institution.

**Table 1:** Description of the participants.

	**Computer Science**	**Economics**	**Exacts**	**Environmental**	**Law**	**Energy**	**Industrial**	**Humanities**	**Total group**
Teachers	16	27	33	22	36	25	26	18	203
Students	303	592	138	836	127	125	293	361	2,775
Total department	319	619	171	858	163	150	319	379	2,978

In relation to the professional category of the participating teachers, they are mostly assistants, followed by adjuncts II and III, and to a lesser extent, holders II and III. On the other hand, in terms of non-university teaching experience, the average is 6 years, ranging from 0 to 45 years compared to university teaching experience, whose average is 10 years.

### Instrument

An ad *hoc* online questionnaire is applied through the online survey tool, Survey Monkey, in its two versions, EMICOG-teachers (García-Martín et al., 2019a; García-Martín and García-Sánchez, 2020) and EMICOG-students (García-Martín et al., 2019b).

The EMICOG is previously validated and with the study samples, with KLM sampling adequacy data (>0.01), indicating McDonald's omega compound reliabilities above 0.90 as well as Cronbach's alpha internal consistencies above 0.85; with a mean variance extracted >0.50 (convergent validity); the discriminant validity (square root of the mean variance extracted) is higher than the intercorrelations between the factors, as expected. For this reason, the construct validity is adequate with indices above what is desirable, like the rest of the indicators. Likewise, the confirmatory factor analysis (CFA) provides adequate measures, in accordance with what is recommended for a CFA, obtaining an NFI (Normed Fix Index), a TLI (Tucker-Lewis Index) and a CFI (Comparative Fit Index), above of 0.90 (as recommended); and with an RMSEA (Root Mean Square Error of Approximation), below 0.08 (as recommended).

The EMICOG consists of four sections: (i) demographic data; (ii) the level of knowledge about the mainstreaming of the ARC; (iii) the instructional strategies used to teach and cross-evaluate ARC and (iv) the textual genres used to teach and cross-evaluate ARC. On the one hand, in relation to the instructional strategies used, in the different type subjects, both to teach and to evaluate advanced reading comprehension or critical reading, the extraction of main ideas, the establishment of relationships between the ideas of some readings are examined. With the previous knowledge, the development of conclusions and inferences not explicit in the readings, the application of solutions suggested in the readings to other aspects, the explanation of the content of some reading and the recovery of the information previously read and its use to instruct. On the other hand, in the case of textual genres used for the teaching and evaluation of ARC, argumentative genres, comparison and contrast, essays, literary analysis, bibliographic reviews, cause-effect or problem-solution, definitions and reviews on the state of the question.

The questions included in the survey were of the Likert type, with five response options (see complete survey for teachers in García-Martín and García-Sánchez, 2020; that of students is very similar with the adapted questions). The duration of completion of the same was 30–40 mins. The responses were made through the Survey Monkey platform. Participations were requested on the occasion of the institutional semester tests of generic competencies in four waves, with all students of all grades participating. The request for participation came from the direction of the Center for Teaching Excellence and the Academic Vice-Rector's Office, urging their participation, both for all teachers and all students. The completion was carried out during the 4 weeks of application of the generic skills tests on the Survey Monkey platform, a professional survey, open 24 hours a day, 7 days a week. After voluntarily accepting to participate, and giving their informed consent, they completed the surveys. The advantages of Survey Monkey are many, registering all the responses, partial or complete, the start and end time, the potential for simultaneous response of thousands of participants, nested response options, among others. The order followed by the instruments was the same for all, according to the description of the different scales described.

### Design and Procedure

An exploratory-descriptive design is followed that is materialized in the application of the two versions (teachers and students) of an ad *hoc* online questionnaire, the EMICOG, which analyses the cognitive strategies and textual genres used in teaching and in cross-sectional evaluation of CRA or advanced reading comprehension, from a double perspective.

### Analysis of Data

In the first place, descriptive analysis of the participating teachers and students are carried out, as well as the verification of the normality of the variables (asymmetry and kurtosis), evidencing that these are normally distributed, so it is appropriate to carry out the parametric analysis. Immediately, reliability and validity analysis of the instrument were carried out in its two modalities (item-scale internal consistency, Cronbach's alphas, composite reliability, extracted mean variance, discriminant validity), using exploratory factor analysis with SPSS v26 (García-Martín et al., 2019a,b) and confirmatory with AMOS v26 with Gaskination's StatWiki plugins (http://statwiki.gaskination.com/index.php?title=Plugins) the Pattern Matrix Model Builder from the pattern matrices were used; as well as the Model Fit Measures, the Validity and Reliability Test and other functionalities. For the calculation of the composite reliabilities, extracted mean variances, convergent and discriminant validity, with Excel from the factor matrices, too. Subsequently, multivariate analysis is carried out based on the general linear model (GLM), considering the department as the grouping variable, the demographic variable common to the two versions of the instrument, and as dependent the rest of the measures on the instruction and evaluation of the critical reading or advanced reading comprehension, through the SPSS version 26, which shows statistically significant differences with high effect sizes.

## Results

As indicated above, the multivariate contrasts (λWilks = 0.003; *F* = 1,277; *p* = 0.001; η^2^ =0.523) of the analysis of variance (ANOVA), carried out through the General Linear Model (GLM), show statistically significant differences with large effect sizes when the department to which the participants are assigned is considered as a grouping variable. We can see the inter-subject effects of professors in the Table 2 for the teaching and assessment of the advanced reading comprehension.

**Table 2:** Tests of the inter-subject effects of the professors on the instructional variables in ARC considering the department as a grouping variable.

**Department**	**Computer Science**	**Economics**	**Exacts**	**Environmental**	**Law**	**Energy**	**Industrial**	**Humanities**	***F***	***p***	**η^**2**^**
**Please indicate your level of knowledge … (min 1–max 5)**											
Of the transversal teaching of generic competences of critical reading and textual construction in the subject	4.23 (0.83)	3.48 (0.75)	3.79 (0.78)	4.14 (0.77)	3.82 (0.77)	4.05 (0.50)	3.44 (0.98)	4.06 (1.00)	2.13	0.04	0.10
**Advanced reading comprehension–has ever been used in this subject tasks, techniques or teaching strategies (or instructional) of. (m**í**n 1–max 5)**											
***Strategies and processes***											
**Relate ideas** from some reading with previous knowledge (**WORKED**)	4.31 (0.63)	4.10 (0.77)	3.71 (1.04)	3.93 (1.07)	4.25 (0.65)	4.33 (0.58)	3.56 (1.20)	4.56 (0.51)	2.78	0.01	0.13
**Relate ideas** from some reading with previous knowledge (**EVALUATE**)	4.23 (0.60)	4.05 (0.81)	3.67 (1.05)	4.07 (0.92)	4.29 (0.66)	4.33 (0.58)	3.50 (1.34)	4.56 (0.51)	2.91	0.01	0.14
**Relate ideas** from some reading with previous knowledge (**UTILITY**)	4.46 (0.66)	4.24 (0.77)	3.96 (1.00)	4.43 (0.65)	4.32 (0.61)	4.19 (0.68)	3.67 (1.19)	4.75 (0.45)	2.71	0.01	0.13
***Textual genres***											
Reading of some **argumentative text** (defend ideas, debate, refute, convince, justify) (**WORKED**)	3.69 (0.63)	3.43 (1.17)	3.13 (1.12)	4.07 (0.83)	4.11 (0.99)	3.57 (1.36)	3.39 (1.38)	4.31 (0.60)	2.63	0.01	0.13
Reading of some **argumentative text** (defend ideas, debate, refute, convince, justify) (**EVALUATE**)	3.62 (0.65)	3.38 (1.20)	3.17 (1.13)	4.14 (0.77)	4.07 (1.05)	3.48 (1.33)	3.28 (1.45)	4.25 (0.68)	2.55	0.01	0.12
Reading of some **argumentative text** (defend ideas, debate, refute, convince, justify) (**UTILITY**)	3.77 (0.73)	3.86 (0.85)	3.50 (1.18)	4.36 (0.75)	4.29 (0.81)	3.57 (1.21)	3.22 (1.44)	4.38 (0.72)	3.10	0.00	0.14
Reading of some **comparison and contrast text**: two theories, concepts, stories, authors, figures, preferences (**WORKED**)	3.38 (0.77)	3.29 (1.19)	3.42 (0.97)	3.57 (1.02)	4.00 (0.90)	3.86 (0.96)	3.06 (1.16)	3.88 (0.89)	2.05	0.04	0.10
Reading of some **comparison and contrast text**: two theories, concepts, stories, authors, figures, preferences (**UTILITY**)	3.62 (0.87)	3.71 (0.96)	3.54 (0.93)	4.14 (0.77)	4.25 (0.65)	3.81 (0.98)	3.22 (1.22)	4.06 (0.93)	2.50	0.01	0.12
Reading of an essay (question-answer, admission, answer, scientific, test answers, opinion) (**WORKED**)	4.00 (0.58)	3.57 (1.03)	3.33 (0.96)	3.79 (0.80)	3.86 (1.08)	3.62 (1.12)	2.94 (1.26)	3.94 (0.77)	2.01	0.05	0.10
Reading of an essay (question-answer, admission, answer, scientific, test answers, opinion) (**EVALUATE**)	3.92 (0.64)	3.48 (1.03)	3.38 (0.97)	3.79 (0.80)	3.86 (1.08)	3.62 (1.12)	2.72 (1.18)	4.00 (0.73)	2.82	0.01	0.13
Reading of an essay (question-answer, admission, answer, scientific, test answers, opinion) (**UTILITY**)	4.08 (0.64)	3.76 (0.83)	3.50 (1.02)	3.93 (0.83)	4.07 (0.94)	3.71 (1.19)	3.11 (1.37)	4.19 (0.83)	2.11	0.04	0.10
Reading of some **analysis text** (description, literary analysis, process analysis) (**EVALUATE**)	4.00 (0.91)	3.29 (0.96)	3.42 (1.18)	3.57 (1.16)	4.04 (0.79)	3.95 (0.92)	3.11 (1.37)	3.56 (1.26)	2.06	0.04	0.10
Reading of some **analysis text** (description, literary analysis, process analysis) (**UTILITY**)	4.15 (0.90)	3.71 (0.90)	3.58 (1.18)	4.00 (0.68)	4.14 (0.65)	4.05 (0.92)	3.06 (1.51)	4.13 (1.09)	2.42	0.02	0.12
***Medium used***											
Reading some text in **blogs** (internet tool) (**WORKED**)	3.08 (1.26)	3.29 (1.10)	2.96 (1.12)	2.71 (1.44)	3.04 (1.07)	2.76 (1.14)	1.78 (1.06)	2.88 (1.03)	2.88	0.01	0.14
Reading some text in **blogs** (internet tool) (**EVALUATE**)	3.00 (1.29)	3.19 (1.12)	2.87 (1.23)	2.64 (1.45)	2.96 (1.04)	2.71 (1.15)	1.89 (1.18)	2.88 (1.03)	2.37	0.02	0.11
Reading some text in **blogs** (internet tool) (**UTILITY**)	3.00 (1.08)	3.67 (0.86)	3.00 (1.22)	3.07 (1.39)	3.25 (0.89)	2.81 (1.12)	2.22 (1.17)	3.00 (1.10)	2.60	0.01	0.12
Reading some text in **wikis** (internet tool) (**WORKED**)	2.85 (0.99)	2.48 (0.93)	2.83 (1.01)	2.36 (1.55)	2.75 (1.18)	2.24 (1.18)	1.61 (1.04)	2.50 (1.03)	2.67	0.01	0.13
Reading some text in **wikis** (internet tool) (**EVALUATE**)	2.77 (0.83)	2.38 (1.02)	2.83 (1.01)	2.29 (1.54)	2.75 (1.18)	2.24 (1.18)	1.56 (1.04)	2.38 (0.89)	2.91	0.01	0.14
Reading some text in **wikis** (internet tool) (**UTILITY**)	3.00 (1.16)	2.86 (1.01)	3.00 (0.98)	2.86 (1.51)	3.00 (1.02)	2.24 (1.14)	2.11 (1.18)	2.69 (1.20)	2.32	0.02	0.11
Readings are preferably done in **digital format** (Word, PDF, eBook, ePub) (**WORKED**)	3.46 (0.78)	3.43 (1.03)	3.83 (0.87)	3.57 (0.76)	3.82 (0.95)	4.05 (0.59)	3.44 (1.20)	3.37 (0.89)	2.00	0.05	0.10
Readings are preferably done in **digital format** (Word, PDF, eBook, ePub) (**EVALUATE**)	3.46 (0.78)	3.38 (1.12)	3.83 (0.87)	3.50 (0.86)	3.79 (0.969	4.00 (0.63)	3.33 (1.28)	3.37 (0.89)	2.86	0.01	0.13

*Only statistically significant variables are included*.

The inter-subject contrasts effects of the multivariate analyzes, referring to the students, are included in detail in Table 3.

**Table 3:** Tests of the inter-subject effects of the students in the instructional variables in ARC considering the department as a grouping variable.

**Department**	**Computer Science**	**Economics**	**Exacts**	**Environmental**	**Law**	**Energy**	**Industrial**	**Humanities**	***F***	***p***	**η^**2**^**
**Please indicate your level of knowledge … (min 1–max 5)**											
of the **SaberPro tests** on critical reading	3.14 (0.74)	3.23 (0.89)	3.14 (0.83)	3.17 (0.74)	3.31 (0.79)	3.10 (0.68)	3.22 (0.82)	3.41 (0.78)	2.60	0.00	0.01
of the **evaluation of critical reading** in the subject	3.24 (0.70)	3.41 (0.83)	3.21 (0.82)	3.32 (0.74)	3.43 (0.83)	3.36 (0.65)	3.39 (0.79)	3.58 (0.81)	4.27	0.00	0.02
of the **evaluation of textual construction** in the subject	3.15 (0.74)	3.34 (0.83)	3.24 (0.82)	3.32 (0.78)	3.44 (0.76)	3.23 (0.73)	3.36 (0.80)	3.60 (0.78)	5.49	0.00	0.02
of the **teaching of critical reading** in the subject	3.25 (0.78)	3.48 (0.80)	3.26 (0.82)	3.34 (0.82)	3.52 (0.80)	3.37 (0.73)	3.46 (0.79)	3.65 (0.84)	5.07	0.00	0.02
of the **transversal teaching** of generic competences of **critical reading and textual construction** in the subject	3.15 (0.81)	3.32 (0.83)	3.26 (0.81)	3.28 (0.77)	3.36 (0.83)	3.20 (0.75)	3.30 (0.80)	3.51 (0.78)	3.32	0.00	0.01
**Indicate your degree of interest. (min 1–max 5)**											
for **critical reading to work** on key aspects of the subject	3.45 (0.85)	3.77 (0.80)	3.53 (0.83)	3.61 (0.81)	3.80 (0.84)	3.48 (0.78)	3.64 (0.78)	3.97 (0.77)	7.26	0.00	0.03
for **critical reading to evaluate** on key aspects of the subject	3.48 (0.86)	3.73 (0.83)	3.59 (0.76)	3.58 (0.80)	3.80 (0.78)	3.47 (0.73)	3.60 (0.78)	3.92 (0.79)	5.90	0.00	0.02
**Advanced reading comprehension-has ever been used in this subject tasks, techniques or teaching strategies (or instructional) of. (m**í**n 1–max 5)**											
***Strategies and processes***											
**Extract the main ideas** of some reading (**WORKED)**	3.53 (1.02)	3.90 (0.90)	3.66 (0.95)	3.65 (0.94)	3.89 (0.95)	3.72 (0.97)	3.83 (0.92)	4.16 (0.85)	9.05	0.00	0.04
**Extract the main ideas** of some reading (**EVALUATE)**	3.47 (1.07)	3.47 (1.07)	3.86 (0.91)	3.59 (0.95)	3.90 (0.91)	3.70 (0.93)	3.82 (0.91)	4.16 (0.83)	10.97	0.00	0.04
**Extract the main ideas** of some reading (**UTILITY)**	3.65 (0.99)	3.90 (0.84)	3.61 (0.99)	3.70 (0.93)	3.99 (0.87)	3.64 (0.96)	3.80 (0.92)	4.23 (0.83)	10.00	0.00	0.04
**Relate ideas** from some reading with **previous knowledge (WORKED)**	3.59 (0.96)	3.92 (0.82)	3.62 (0.88)	3.74 (0.88)	3.98 (0.85)	3.64 (0.95)	3.80 (0.87)	4.20 (0.78)	9.51	0.00	0.04
**Relate ideas** from some reading with **previous knowledge (EVALUATE)**	3.53 (0.99)	3.91 (0.82)	3.54 (0.92)	3.67 (0.88)	3.97 (0.85)	3.58 (0.95)	3.80 (0.89)	4.13 (0.82)	10.14	0.00	0.04
**Relate ideas** from some reading with **previous knowledge (UTILITY)**	3.67 (0.97)	3.91 (0.77)	3.57 (0.96)	3.71 (0.87)	3.94 (0.89)	3.70 (0.88)	3.82 (0.89)	4.18 (0.80)	9.09	0.00	0.04
**Draw conclusions and inferences** not explicit in the readings (**WORKED**)	3.47 (1.00)	3.73 (0.88)	3.48 (1.05)	3.59 (0.90)	3.81 (0.86)	3.58 (0.94)	3.70 (0.92)	4.08 (0.82)	8.62	0.00	0.03
**Draw conclusions and inferences** not explicit in the readings (**EVALUATE**)	3.44 (1.00)	3.73 (0.86)	3.49 (0.99)	3.59 (0.94)	3.80 (0.87)	3.57 (0.95)	3.73 (0.93)	4.10 (0.80)	9.35	0.00	0.04
**Draw conclusions and inferences** not explicit in the readings (**UTILITY**)	3.56 (1.00)	3.73 (0.85)	3.57 (1.01)	3.62 (0.92)	3.86 (0.87)	3.50 (0.94)	3.72 (0.91)	4.08 (0.83)	7.92	0.00	0.03
**Apply solutions** to other aspects, suggested by the readings (**WORKED**)	3.39 (0.99)	3.70 (0.88)	3.50 (0.99)	3.56 (0.90)	3.83 (0.83)	3.55 (0.93)	3.69 (0.90)	4.03 (0.81)	8.54	0.00	0.03
**Apply solutions** to other aspects, suggested by the readings (**EVALUATE**)	3.41 (1.02)	3.68 (0.90)	3.42 (0.98)	3.55 (0.92)	3.82 (0.84)	3.54 (0.96)	3.69 (0.89)	4.01 (0.84)	8.45	0.00	0.03
**Apply solutions** to other aspects, suggested by the readings (**UTILILTY**)	3.50 (0.94)	3.72 (0.86)	3.53 (0.92)	3.58 (0.88)	3.90 (0.80)	3.53 (0.91)	3.65 (0.87)	4.03 (0.77)	8.71	0.00	0.03
**Explain** the basic content of some reading (**WORKED**)	3.53 (0.98)	3.86 (0.85)	3.62 (0.96)	3.66 (0.86)	3.97 (0.82)	3.62 (0.94)	3.83 (0.87)	4.22 (0.74)	11.99	0.00	0.05
**Explain** the basic content of some reading (**EVALUATE**)	3.55 (1.04)	3.82 (0.87)	3.51 (0.97)	3.63 (0.88)	3.98 (0.79)	3.58 (0.95)	3.75 (0.89)	4.18 (0.78)	10.80	0.00	0.04
**Explain** the basic content of some reading (**UTILITY**)	3.65 (0.99)	3.84 (0.80)	3.59 (0.94)	3.65 (0.88)	4.05 (0.81)	3.69 (0.94)	3.81 (0.88)	4.18 (0.88)	11.28	0.00	0.04
Remember without consulting the information previously read (**retrieve**). To consult again later and to recover without consulting to go completing what is not remembered (**WORKED**)	3.34 (1.01)	3.58 (0.87)	3.40 (0.95)	3.52 (0.90)	3.73 (0.86)	3.55 (0.98)	3.60 (0.89)	3.85 (0.83)	5.05	0.00	0.02
Remember without consulting the information previously read (**retrieve**). To consult again later and to recover without consulting to go completing what is not remembered (**EVALUATE**)	3.34 (1.03)	3.60 (0.87)	3.41 (0.99)	3.51 (0.91)	3.72 (0.89)	3.57 (0.97)	3.59 (0.88)	3.85 (0.85)	5.26	0.00	0.02
Remember without consulting the information previously read (**retrieve**). To consult again later and to recover without consulting to go completing what is not remembered (**UTILITY**)	3.44 (0.97)	3.60 (0.85)	3.48 (0.97)	3.55 (0.85)	3.73 (0.87)	3.39 (1.00)	3.59 (0.82)	3.90 (0.79)	5.47	0.00	0.02
***Textual genres***											
Reading of some **argumentative text** (defend ideas, debate, refute, convince, justify) (**WORKED**)	3.36 (1.05)	3.71 (0.93)	3.43 (1.04)	3.53 (1.01)	3.93 (0.92)	3.54 (1.07)	3.63 (0.94)	4.12 (0.80)	11.86	0.00	0.05
Reading of some **argumentative text** (defend ideas, debate, refute, convince, justify) (**EVALUATE**)	3.33 (1.07)	3.69 (0.95)	3.43 (1.01)	3.51 (1.01)	3.92 (0.90)	3.46 (1.08)	3.58 (0.99)	4.08 (0.819	11.16	0.00	0.04
Reading of some **argumentative text** (defend ideas, debate, refute, convince, justify) (**UTILITY**)	3.39 (1.10)	3.74 (0.87)	3.48 (1.01)	3.58 (0.95)	3.94 (0.83)	3.52 (1.06)	3.61 (0.92)	4.14 (0.80)	11.84	0.00	0.05
Reading of some **comparison and contrast text**: two theories, concepts, stories, authors, figures, preferences (**WORKED**)	3.30 (1.04)	3.64 (0.94)	3.37 (0.95)	3.50 (0.90)	3.81 (0.96)	3.51 (1.00)	3.63 (0.90)	4.06 (0.84)	11.47	0.00	0.04
Reading of some **comparison and contrast text**: two theories, concepts, stories, authors, figures, preferences (**EVALUATE**)	3.31 (1.02)	3.64 (0.94)	3.33 (0.95)	3.49 (0.97)	3.81 (0.95)	3.53 (1.02)	3.62 (0.93)	4.04 (0.78)	11.47	0.00	0.04
Reading of some **comparison and contrast text**: two theories, concepts, stories, authors, figures, preferences (**UTILITY**)	3.43 (1.06)	3.71 (0.87)	3.51 (0.90)	3.54 (0.90)	3.87 (0.86)	3.50 (0.98)	3.61 (0.88)	4.09 (0.80)	11.41	0.00	0.04
Reading of an **essay** (question-answer, admission, answer, scientific, test answers, opinion) (**WORKED**)	3.36 (1.07)	3.67 (0.91)	3.40 (0.97)	3.53 (0.94)	3.83 (0.84)	3.48 (0.99)	3.57 (0.91)	3.93 (0.84)	6.76	0.00	0.03
Reading of an **essay** (question-answer, admission, answer, scientific, test answers, opinion) (**EVALUATE**)	3.38 (1.04)	3.65 (0.93)	3.39 (0.96)	3.50 (0.95)	3.78 (0.93)	3.44 (1.04)	3.63 (0.89)	3.88 (0.84)	6.66	0.00	0.03
Reading of an **essay** (question-answer, admission, answer, scientific, test answers, opinion) **(UTILITY**)	3.45 (1.01)	3.69 (0.87)	3.45 (0.92)	3.59 (0.88)	3.89 (0.81)	3.50 (0.99)	3.61 (0.86)	4.02 (0.81)	8.82	0.00	0.03
Reading of some **analysis text** (description, literary analysis, process analysis) (**WORKED**)	3.42 (1.07)	3.68 (0.90)	3.41 (0.89)	3.55 (0.89)	3.83 (0.86)	3.56 (1.00)	3.64 (0.87)	4.00 (0.84)	8.35	0.00	0.03
Reading of some **analysis text** (description, literary analysis, process analysis) (**EVALUATE**)	3.38 (1.05)	3.68 (0.93)	3.40 (0.95)	3.50 (0.92)	3.83 (0.87)	3.53 (1.03)	3.64 (0.91)	4.01 (0.82)	9.52	0.00	0.04
Reading of some **analysis text** (description, literary analysis, process analysis) (**UTILITY**)	3.44 (1.03)	3.71 (0.84)	3.51 (0.92)	3.54 (0.88)	3.86 (0.89)	3.45 (1.00)	3.61 (0.85)	4.02 (0.80)	9.92	0.00	0.04
Reading a **bibliographic review** (**WORKED**)	3.21 (1.08)	3.50 (1.04)	3.36 (0.98)	3.43 (0.99)	3.62 (0.98)	3.43 (1.03)	3.49 (1.01)	3.89 (0.89)	6.31	0.00	0.03
Reading a **bibliographic review** (**EVALUATE**)	3.16 (1.14)	3.52 (1.03)	3.30 (1.01)	3.43 (1.01)	3.70 (0.96)	3.47 (1.03)	3.49 (1.04)	3.90 (0.87)	6.91	0.00	0.03
Reading a **bibliographic review** (**UTILITY**)	3.32 (1.05)	3.55 (1.00)	3.47 (0.99)	3.46 (0.96)	3.70 (0.90)	3.43 (1.04)	3.56 (0.96)	3.97 (0.80)	7.69	0.00	0.03
Reading some **cause-effect/problem-solution text (WORKED**)	3.36 (0.87)	3.62 (0.94)	3.40 (1.02)	3.50 (0.94)	3.68 (0.90)	3.58 (0.93)	3.65 (0.93)	3.82 (0.90)	4.67	0.00	0.02
Reading some **cause-effect/problem-solution text (EVALUATE**)	3.38 (1.12)	3.62 (0.94)	3.34 (1.02)	3.47 (0.97)	3.72 (0.91)	3.62 (0.97)	3.66 (0.92)	3.82 (0.90)	5.81	0.00	0.02
Reading some **cause-effect/problem-solution text (UTILITY**)	3.47 (1.04)	3.64 (0.88)	3.47 (0.88)	3.53 (0.91)	3.80 (0.88)	3.55 (0.98)	3.66 (0.92)	3.91 (0.83)	5.50	0.00	0.02
Reading some **definition text (WORKED**)	3.46 (1.03)	3.74 (0.85)	3.49 (0.91)	3.60 (0.86)	3.86 (0.89)	3.63 (1.03)	3.73 (0.94)	3.97 (0.94)	6.65	0.00	0.03
Reading some **definition text (EVALUATE**)	3.44 (1.08)	3.74 (0.87)	3.51 (0.90)	3.59 (0.87)	3.80 (0.93)	3.57 (1.04)	3.70 (0.93)	3.94 (0.84)	6.40	0.00	0.03
Reading some **definition text (UTILITY**)	3.49 (1.06)	3.74 (0.83)	3.59 (0.84)	3.63 (0.86)	3.82 (0.91)	3.51 (1.03)	3.73 (0.90)	3.97 (0.79)	6.06	0.00	0.02
Reading of a text to **review the state of the question** (topic, theory, approach, scientific, antecedents, previous experiences, successful solutions) (**WORKED**)	3.37 (1.01)	3.61 (0.89)	3.42 (0.86)	3.52 (0.91)	3.72 (0.91)	3.58 (1.01)	3.57 (0.98)	3.97 (0.80)	6.89	0.00	0.03
Reading of a text to **review the state of the question** (topic, theory, approach, scientific, antecedents, previous experiences, successful solutions) (**EVALUATE**)	3.42 (1.04)	3.61 (0.92)	3.43 (0.88)	3.52 (0.89)	3.72 (0.90)	3.55 (0.98)	3.60 (0.98)	4.00 (0.81)	7.40	0.00	0.03
Reading of a text to **review the state of the question** (topic, theory, approach, scientific, antecedents, previous experiences, successful solutions) (**UTILITY**)	3.48 (1.02)	3.66 (0.85)	3.59 (0.80)	3.54 (0.89)	3.83 (0.89)	3.53 (0.99)	3.61 (0.89)	4.02 (0.79)	8.75	0.00	0.03
***Medium used***											
Reading a document in **digital databases (WORKED)**	3.64 (0.96)	3.64 (0.96)	3.38 (1.04)	3.50 (0.98)	3.54 (0.97)	3.70 (1.04)	3.65 (0.92)	3.93 (0.90)	4.91	0.00	0.02
Reading a document in **digital databases (EVALUATE)**	3.53 (1.00)	3.63 (0.99)	3.32 (0.98)	3.52 (0.97)	3.57 (1.00)	3.70 (1.06)	3.62 (0.98)	3.96 (0.86)	5.25	0.00	0.02
Reading a document in **digital databases (UTILITY)**	3.71 (0.94)	3.69 (0.98)	3.45 (0.94)	3.61 (0.92)	3.69 (0.87)	3.65 (0.97)	3.64 (0.92)	4.01 (0.79)	4.94	0.00	0.02
Readings are preferably done in **traditional format** (paper, notes, articles, books) **(WORKED**)	3.29 (1.01)	3.52 (0.98)	3.58 (0.92)	3.52 (0.94)	3.80 (0.89)	3.71 (0.87)	3.65 (0.99)	3.78 (0.96)	4.23	0.00	0.02
Readings are preferably done in **traditional format** (paper, notes, articles, books) **(EVALUATE**)	3.30 (1.08)	3.56 (0.99)	3.52 (0.94)	3.52 (0.94)	3.74 (0.95)	3.74 (0.87)	3.60 (0.99)	3.84 (0.92)	4.49	0.00	0.02
Readings are preferably done in **traditional format** (paper, notes, articles, books) **(UTILITY**)	3.43 (1.07)	3.59 (0.95)	3.58 (0.93)	3.63 (0.86)	3.79 (0.86)	3.64 (0.86)	3.58 (0.87)	3.89 (0.90)	3.70	0.00	0.02
Readings are preferably done in **digital format** (Word, PDF, eBook, ePub) **(WORKED**)	3.67 (0.94)	3.81 (0.90)	3.54 (0.95)	3.68 (0.88)	3.80 (0.94)	3.72 (0.93)	3.84 (0.88)	4.04 (0.86)	4.41	0.00	0.02
Reading some text in **blogs** (internet tool) (**WORKED**)	3.64 (0.94)	3.80 (0.88)	3.41 (1.00)	3.63 (0.90)	3.76 (0.92)	3.72 (0.91)	3.77 (0.87)	4.04 (0.78)	5.64	0.00	0.02
Reading some text in **blogs** (internet tool) (**EVALUATE**)	3.72 (0.90)	3.80 (0.87)	3.65 (0.80)	3.69 (0.88)	3.84 (0.90)	3.74 (0.89)	3.81 (0.86)	4.06 (0.78)	4.36	0.00	0.02
**Audiobooks or video documents** are used (**WORKED**)	3.20 (1.19)	3.19 (1.20)	2.95 (1.19)	3.17 (1.16)	3.12 (1.16)	3.37 (1.12)	3.18 (1.22)	3.46 (1.17)	2.15	0.02	0.01
**Audiobooks or video documents** are used (**EVALUATE**)	3.16 (1.24)	3.17 (1.18)	2.96 (1.23)	3.15 (1.15)	3.13 (1.19)	3.37 (1.13)	3.23 (1.21)	3.49 (1.17)	2.53	0.00	0.01

*Only statistically significant variables are included*.

**Figure 1:**
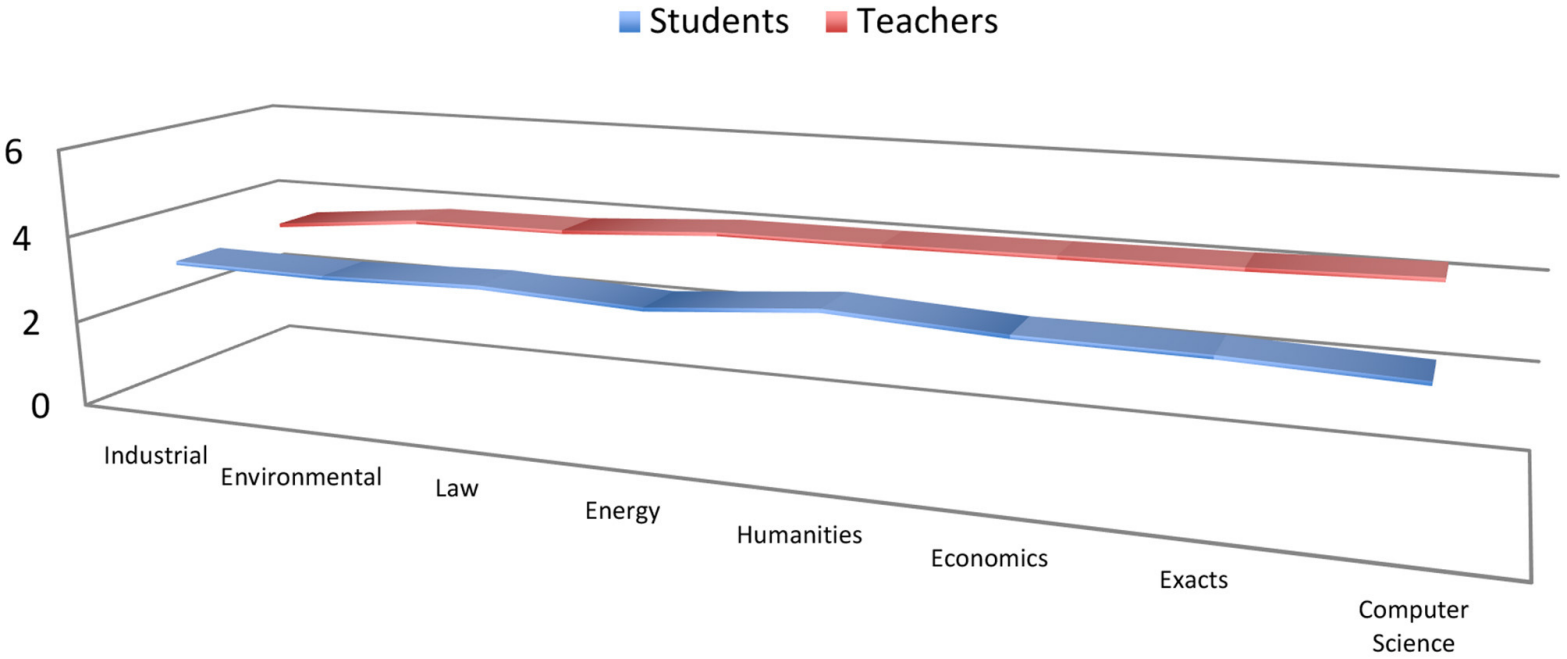
Degree of knowledge about the transversal teaching of the ARC.

For a better understanding of the results, different figures are included below that provide a global vision. In this sense, as can be seen in Figure 1, when examining the variable of degree of knowledge that teachers and students have about the cross-sectional teaching of the ARC, in the inter-subject tests it is shown that the scores obtained by teachers are higher than students regardless of the department to which they belong (e.g., M_Humanities teachers_ = 4.06 vs. M_Humanities students_ = 3.51; *p* ≤ 0.01; e.g., M_Environmental teachers_ = 3.79 vs. M_Environmental teachers_ = 3.26; *p* ≤ 0.01).

### Cognitive Strategies for Teaching and Cross-Evaluating ARC

**Figure 2:**
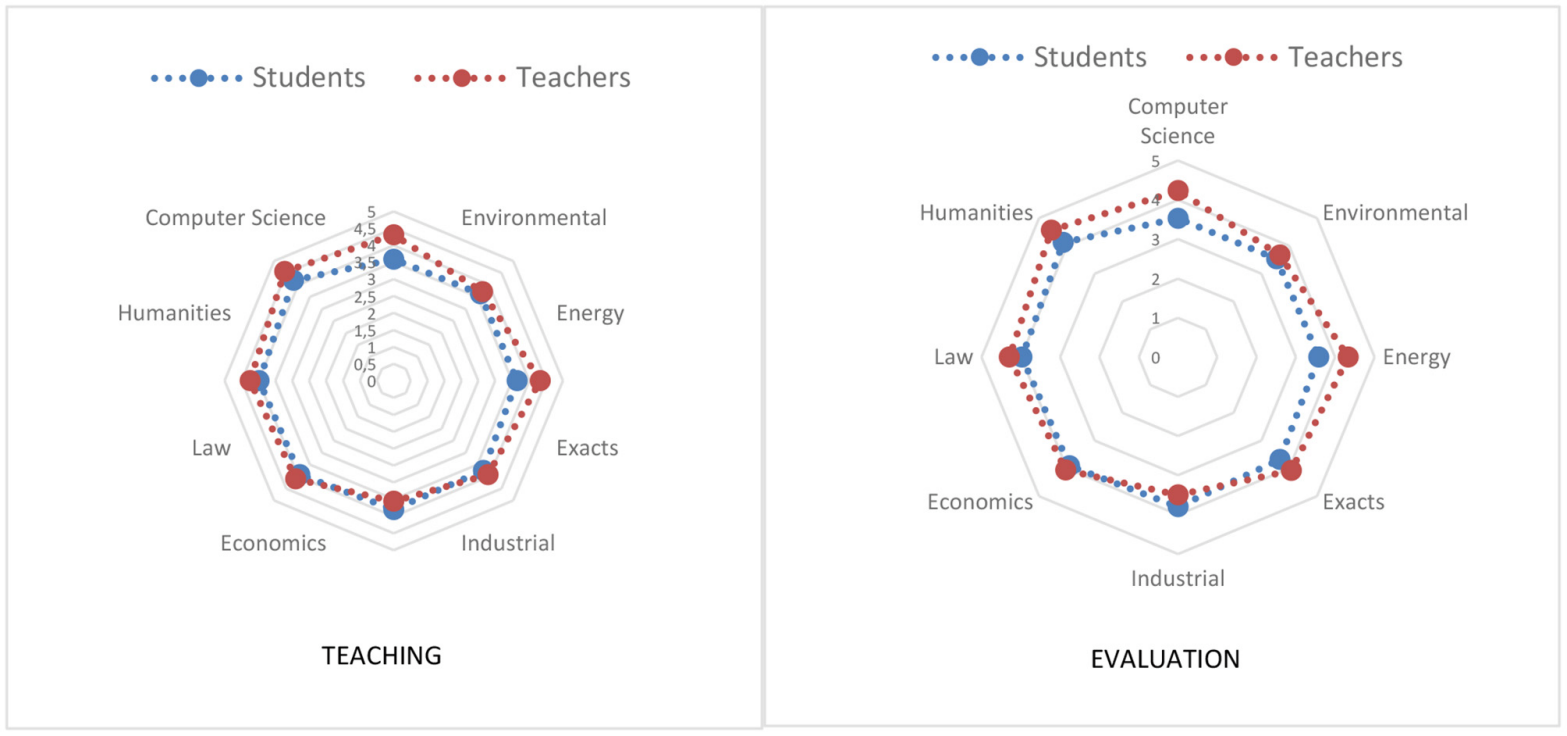
Use of the cognitive strategy for teaching and evaluation ARC.

When the results obtained in the cognitive strategies for teaching and cross-evaluating ARC are compared, by teachers and students from different departments, several trends are observed. On the one hand, as can be seen in Figure 2, the trend described above is maintained, that is, teachers show higher scores than students. Likewise, it is observed that, of the cognitive strategies examined: the extraction of main ideas, the establishment of relationships between the ideas of some readings with previous knowledge, the development of conclusions and inferences not explicit in the readings, the application of solutions suggested in the readings to other aspects, the explanation of the content of some reading and retrieval of previously read information use for instructing. Statistically significant differences are only shown in establishing relationships between the ideas of the readings with previous knowledge, perceiving that the use of this is lower among Computer Science students followed by those of Environmental, Energy, Exact, Industrial, Economic, Law and Humanities both in teaching and in evaluation. On the other hand, in the case of professors, the order is modified starting with those of the Industrial department and followed by those of Environmental, Economics, Exact, Informatics, Law, Energy and Humanities.

### Textual Genres to Teach and Cross-Evaluate ARC

**Figure 3:**
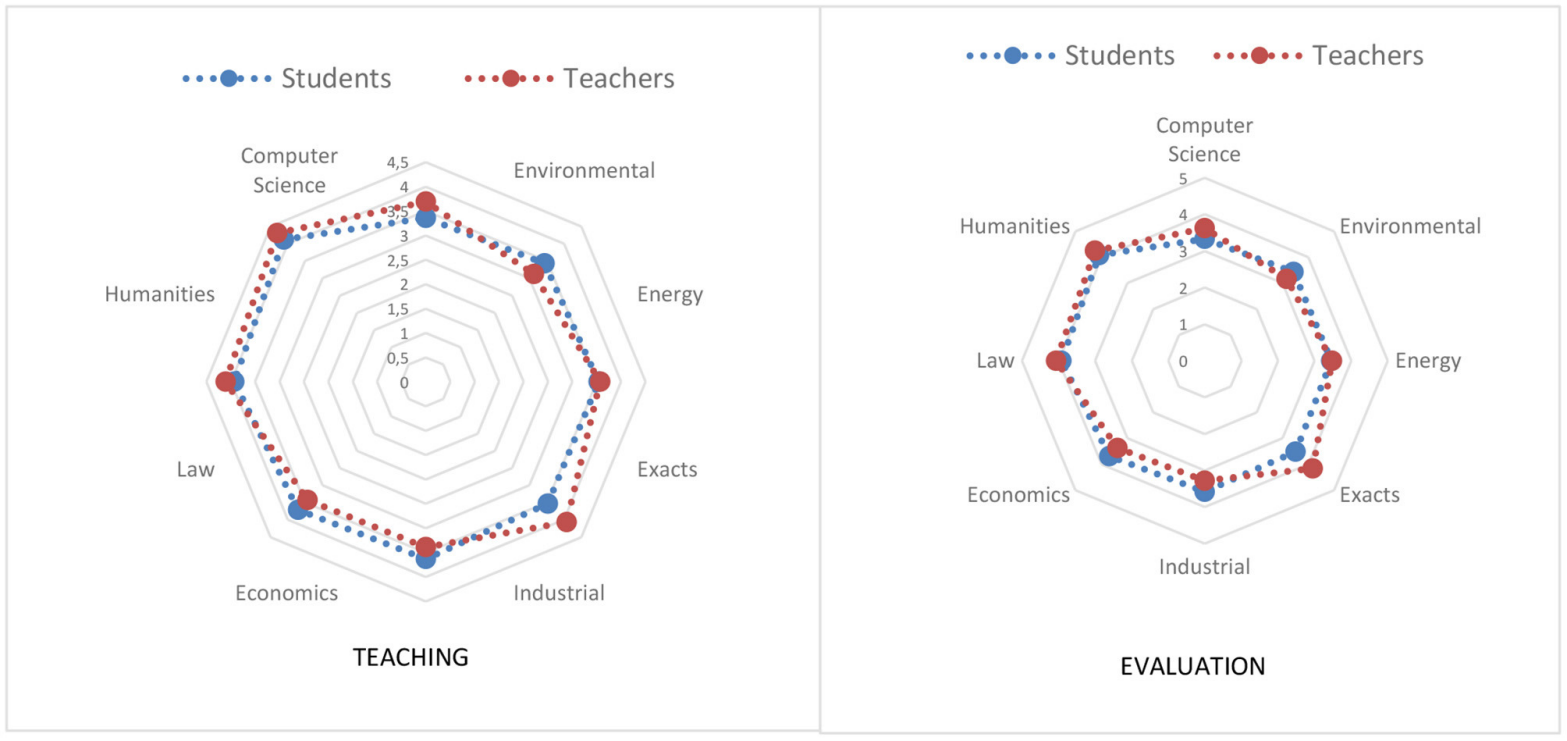
Teaching and evaluation of ARC through an argumentative text.

**Figure 4:**
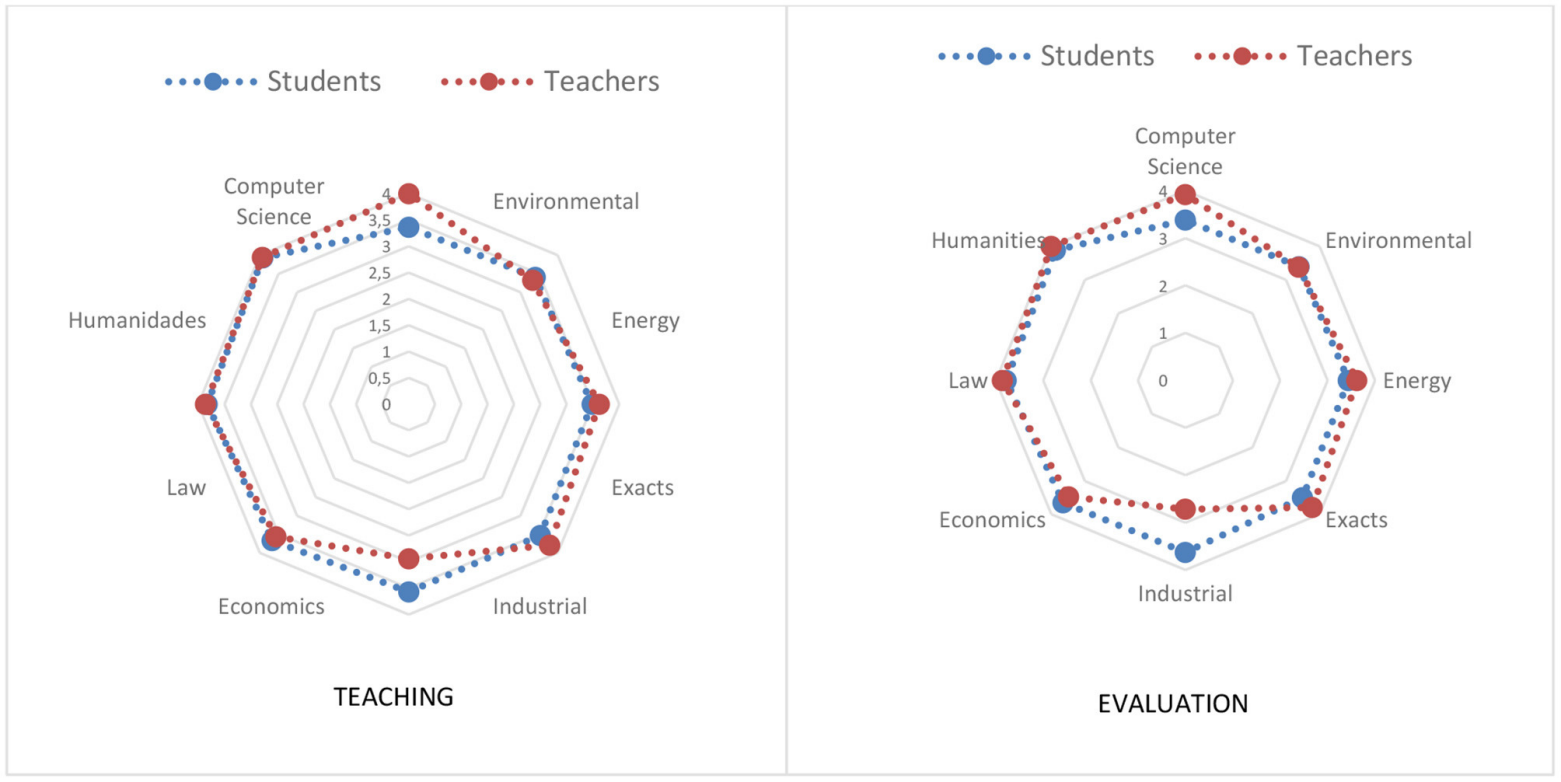
Teaching and evaluation of ARC through an essay text.

Regarding the textual genres examined to teach and cross-evaluate ARC, statistically significant differences are only evidenced in the use of argumentative texts (see Figure 3) and in trials (see Figure 4), not being observed in comparison and contrast, literary analysis, bibliographic reviews, cause-effect or problem-solution, definitions, and reviews on the state of the question.

In this sense, as can be seen in Figure 3, the trend shown above is partially maintained, in the case of argumentative texts, that is, teachers achieve higher scores than students except for those assigned to the departments of Environmental, Industrial and Economic. In addition, in the case of students there is a trend of greater use depending on the department [Computer Science, Environmental, Exact and/or Energy (these last two departments are the only ones whose order is altered), Industrial, Economics, Law and Humanities]. In the same way, in the case of teachers, another clear trend is evident by department [Environmental, Industrial, Economics, Energy, Computer Science, Law and/or Exact (these last two are the only departments whose order is altered) and Humanities].

As shown in Figure 4, the trend evidenced in the argumentative texts is maintained in the essays, that is, the teachers show higher scores than the students, except for the Environmental, Industrial and Economic departments. In addition, when examining both their use when teaching and cross-evaluating the ARC, in the case of students a trend of greater use is observed by department (Computer Science, Environmental, Energy, Exact, Industrial, Economics, Law and Humanities) and of the in the same way, in the case of teaching staff, there is another trend by department [Industrial, Natural Sciences, Economics, Energy, Civil, Law, Humanities and/or Informatics (these last two are the only departments whose order is altered)].

## Discussion and Conclusions

This reaches the initial purpose of analyzing the cognitive strategies and the textual genres used in teaching and in the transversal evaluation of critical reading or advanced reading comprehension, in a Latin American university, on the Coast, from a double perspective: that of the teaching staff and that of the students. Likewise, the hypotheses are confirmed, on the one hand, in relation to the first, it is evidenced that teachers show greater knowledge of cognitive strategies and textual genres to teach and evaluate ARC than students, regardless of the department of assignment. In the same way, the second hypothesis is confirmed since differential patterns are observed in the use of cognitive strategies and textual genres to teach and evaluate ARC by teachers and students, depending on the department to which they are assigned. In this sense, it is not surprising that it is the professors assigned to the Humanities department that show a greater use of cognitive strategies and textual genres for teaching and cross-sectional evaluation of the ARC and that it is those of Computer Science that exhibit the least.

However, it is true that the extrapolation of the results obtained to the entire Colombian higher education territory is questionable, given that the data collected comes from the same higher education institution. That is why the replication of this study in other Colombian universities is recommended in order to verify or refute the trends in the use of cognitive strategies and textual typologies used both to teach and to cross-evaluate ARC. This approach is in line with the most recent advances in the field of literacy across content/discipline/curriculum and the need for its empowerment and deployment in the university (Miller et al., 2018; Scott and Washburn, 2018; Van Ockenburg et al., 2019). The peculiar characteristics of the Latin American university refer to the need to advance in the knowledge of the cognitive strategies and the textual genres used for the teaching and evaluation of the generic competence of ARC, in the different subjects of the different careers and studies. The identification of these, as well as their adaptation and use in the different fields of knowledge, seems mandatory, for the improvement of educational quality and learning results, both in generic competences and in the rest of the subjects. In this sense, there are several emerging research lines that require more evidence, such as the specificity in each professional field, the key personal and institutional variables, as well as other desirable measures that should be implemented in the future, including the deployment of studies with other methodologies different from self-report, such as the observation or contribution of evidence and learning results and experiences validated empirically or scientifically (Valero et al., 2015; Uttl et al., 2017; Zhao and Zhang, 2017; Graham et al., 2018; CEEDAR, 2021; USOE, 2021).

It seems reasonable to include various performances in the future. On the one hand, the implementation of longitudinal studies and the inclusion of digital literacy seem obligatory, which would allow us to know more precisely the nature and role of generic competences, specifically advanced reading comprehension, as well as its impact on learning. rest of psychoeducational competencies and variables (García-Martín and García-Martín, 2021; Robledo-Ramón and García-Gutiérrez, 2021). In addition, the need for exploratory studies on the training that teachers and students, from different fields and careers, must receive and promote to implement the mainstreaming of advanced reading comprehension, such as online workshops and webinars (Hatlevik and Hatlevik, 2018; Daumiller et al., 2019; He et al., 2020). It is also evident that the development of strategies, techniques and instructional procedures for cross-sectional evaluation and teaching, as well as virtual ones, must be contemplated, both for students and teachers (López et al., 2021), the construction of systems of evaluation and instruction, among others (Utama et al., 2020). Another action could involve the design of forms on advanced reading comprehension strategies, such as the case of checklists or others (Mohamadi, 2018; Yunusa and Umar, 2021). Finally, the implementation of online records and protocols for the instruction and cross-sectional evaluation of advanced reading comprehension and other generic and specific competences, as has been done with the citizen with lateral reading and checking fakes while reading information from various subjects and fields (Brodsky et al., 2021; Cabrera et al., 2021; Fandiño-Parra et al., 2021).

### Quality Assessment

In short, it is therefore a study that allows optimizing this transversal competence in students that, in turn, favors the performance of these in the *Saber Pro* tests of the reading module, to which everyone who wants to opt for a degree in Colombian Higher Education, in accordance with the guidelines of the Colombian Institute for the Evaluation of Education (Calderón et al., 2021) and thus reducing the chances of university student desertion (CUC, 2018).

## Data Availability Statement

The raw data supporting the conclusions of this article will be made available by the authors, without undue reservation.

## Ethics Statement

The studies involving human participants were reviewed and approved by Comité De Ética De La Universidad De La Costa. The patients/participants provided their written informed consent to participate in this study.

## Author Contributions

J-NG-S and JG-M: implemented the study. J-NG-S and JG-M conducted the statistical analyses and drafted the manuscript. Both authors performed substantial contributions to the conception of the study, reviewed the manuscript critically for relevant intellectual content, and approved the submitted version. All authors contributed to the article and approved the submitted version.

## Funding

Competitive Research Project evidence CONV-ÍNDEX Ref. 13-2018, Code of Project INV. 150-01-007-13. Assessed by the ACAC (MEN Colombia).

## Conflict of Interest

The authors declare that the research was conducted in the absence of any commercial or financial relationships that could be construed as a potential conflict of interest.

## Publisher's Note

All claims expressed in this article are solely those of the authors and do not necessarily represent those of their affiliated organizations, or those of the publisher, the editors and the reviewers. Any product that may be evaluated in this article, or claim that may be made by its manufacturer, is not guaranteed or endorsed by the publisher.
